# Mendelian Randomization Analysis Identifies Inverse Causal Relationship between External Eating and Metabolic Phenotypes

**DOI:** 10.3390/nu16081166

**Published:** 2024-04-13

**Authors:** Yanina Timasheva, Zhanna Balkhiyarova, Diana Avzaletdinova, Tatyana Morugova, Gulnaz F. Korytina, Arie Nouwen, Inga Prokopenko, Olga Kochetova

**Affiliations:** 1 Institute of Biochemistry and Genetics, Ufa Federal Research Centre of Russian Academy of Sciences, Ufa 450054, Russia; guly_kory@mail.ru (G.F.K.); olga_mk78@mail.ru (O.K.); 2 Department of Medical Genetics and Fundamental Medicine, Bashkir State Medical University, Ufa 450008, Russia; hyppocrat@mail.ru; 3 Section of Statistical Multi-Omics, Department of Clinical & Experimental Medicine, School of Biosciences & Medicine, University of Surrey, Guildford GU2 7XH, UK; z.balkhiiarova@surrey.ac.uk (Z.B.); i.prokopenko@surrey.ac.uk (I.P.); 4 Department of Endocrinology, Bashkir State Medical University, Ufa 450008, Russia; tmorugova@yandex.ru; 5 Department of Biology, Bashkir State Medical University, Ufa 450008, Russia; 6 Department of Psychology, Middlesex University, London NW4 4BT, UK; a.nouwen@mdx.ac.uk

**Keywords:** Mendelian randomization, eating behaviour, DEBQ, genetic predictors

## Abstract

Disordered eating contributes to weight gain, obesity, and type 2 diabetes (T2D), but the precise mechanisms underlying the development of different eating patterns and connecting them to specific metabolic phenotypes remain unclear. We aimed to identify genetic variants linked to eating behaviour and investigate its causal relationships with metabolic traits using Mendelian randomization (MR). We tested associations between 30 genetic variants and eating patterns in individuals with T2D from the Volga-Ural region and investigated causal relationships between variants associated with eating patterns and various metabolic and anthropometric traits using data from the Volga-Ural population and large international consortia. We detected associations between *HTR1D* and *CDKAL1* and external eating; between *HTR2A* and emotional eating; between *HTR2A*, *NPY2R*, *HTR1F*, *HTR3A*, *HTR2C*, *CXCR2*, and T2D. Further analyses in a separate group revealed significant associations between metabolic syndrome (MetS) and the loci in *CRP*, *ADCY3*, *GHRL*, *CDKAL1*, *BDNF*, *CHRM4*, *CHRM1*, *HTR3A*, and *AKT1* genes. MR results demonstrated an inverse causal relationship between external eating and glycated haemoglobin levels in the Volga-Ural sample. External eating influenced anthropometric traits such as body mass index, height, hip circumference, waist circumference, and weight in GWAS cohorts. Our findings suggest that eating patterns impact both anthropometric and metabolic traits.

## 1. Introduction

Individual eating behaviour can manifest in different ways and may lead to the excessive consumption of calorie-dense foods, contributing to weight gain, obesity, and type 2 diabetes (T2D). Being overweight plays a substantial role in the progression of T2D and can reduce the effectiveness of treatment [1]. Insulin resistance tends to increase with increasing adipose tissue, especially in the visceral region, and eating disorders significantly contribute to obesity [2]. The impact of eating behaviour on T2D risk can be further compounded by the presence of other risk factors, such as family history and genetic predisposition [3].

Additionally, a concept related to eating behaviour known as food reinforcement explores how much effort an individual is willing to exert to obtain tasty food [4]. Research indicates that there are individual differences, with obese individuals showing a greater incidence of food addiction and greater attachment to food than non-obese individuals [5]. Understanding the functions of the food reward system, particularly its regulation by neurotransmitters, is crucial role in addressing potential food addiction, overeating, and obesity [6].

Ongoing debates surround how brain dysfunction impacts metabolic disorders, including the role of the cholinergic pathway. Neuronal nicotinic cholinergic receptors influence central and peripheral mechanisms related to eating behaviour and energy balance [7,8]. Variations in the gamma-aminobutyric acid receptor subunit alpha-2 (*GABRA2*) and GRB2-associated binding protein 2 (*GAB2*) genes are linked to behavioural problems, substance use, drinking behaviour, and obesity [9,10,11]. Neuropeptide Y (NPY) and its receptor NPY2R are crucial in regulating appetite control, particularly in the context of obesity and diabetes in the hypothalamus [12]. Genes in the leptin–melanocortin pathway, such as leptin (*LEP*), leptin receptor (*LEPR*), melanocortin-4 receptor (*MC4R*), adenylyl cyclase 3 (*ADCY3*), and brain-derived neurotrophic factor (*BDNF*) genes, are established contributors to obesity and insulin resistance [13]. Additionally, gastric inhibitory polypeptide (GIPR) and ghrelin signalling are critical in food addiction disorders and T2D [14,15].

Oxidative stress and chronic inflammation are believed to be crucial in the pathophysiology of type 2 diabetes (T2D) [16]. Genes such as sirtuin-1 (*SIRT1*), C-reactive protein (*CRP*), C-X-C motif chemokine receptor 2 gene (*CXCR2*), as well as chemokines and interleukins such as interleukin 12 (*IL12A*) and C-X-C motif chemokine ligand 8 (*CXCL8*), are strongly associated with insulin resistance and T2D [17,18]. *CXCL8*, a major adipocytokine, inhibits insulin-induced protein kinase B (AKT) phosphorylation in adipocytes, contributing to insulin resistance [19]. The phosphoinositide 3-kinase (PI3K)/AKT signalling pathway, crucial for proliferation, differentiation, and metabolism, is dysregulated in metabolic disorders, including obesity, diabetes, and insulin resistance [20]. Genome-wide association analyses (GWASs) reveal that mutations in the CDK5 regulatory subunit-associated protein 1-like 1 (*CDKAL1*) gene can impair insulin secretion, elevating T2D risk [21].

Mendelian randomization (MR) is a method that utilizes genetic variants as instrumental variables to discern causality between a modifiable exposure (typically a risk factor) and an outcome (such as a disease or health condition) [22]. By capitalizing on the random assignment of genetic variants at birth, unaffected by environmental influences or reverse causation, MR effectively distinguishes genuine causal relationships from associations uncovered in observational studies [23]. MR not only corroborates the findings of observational research but also identifies adjustable factors that could serve as drug targets and offer insights for shaping public health policies [23]. MR studies in the context of eating behaviour are relatively new but have the potential to provide valuable insights into the causal relationships between genetic variants, eating behaviours, and health outcomes such as T2D [24,25].

GWASs have identified numerous variants linked to metabolic traits, primarily in European populations, limiting the transferability of their results globally. Multi-ancestry studies are vital in understanding genetic contributions to diseases like T2D [26]. The population of the Volga-Ural region, with its blend of European and Asian influences, presents a promising genetic research opportunity due to its multi-ethnic composition shaped by migration between Europe and Asia. Recent studies indicate that among young adults residing in the Volga-Ural region, approximately 13.2% were found to exhibit food addiction as assessed by DEBQ [27]. Additionally, the prevalence of metabolic syndrome (MetS) in this population was reported to be approximately 26.7% [28].

To conclude, it is widely accepted that disordered eating contributes to weight gain, obesity, and T2D. However, the underlying mechanisms linking various eating patterns with certain metabolic phenotypes remain obscure. Our aim was to bridge this gap by identifying genetic variants associated with eating behaviour and to explore the causal relationship between eating behaviour and metabolic traits using an MR approach.

## 2. Materials and Methods

### 2.1. Study Group

The study group included 200 people with type 2 diabetes (T2D) (≥40 years) and 397 healthy participants without diabetes or any other chronic conditions recruited between 2014 and 2022 at the Ufa City Hospital N° 21 and at the Bashkir State Medical University Clinic (Ufa, Russian Federation). The recruitment process for both the T2D and control groups was previously described elsewhere [29,30]. The inclusion criteria for the T2D group were as follows: aged 40 years and older, had a T2D diagnosis established according to WHO criteria (1999–2013) [31], and lacked clinical symptoms of other types of diabetes not related to other participants in the study. The inclusion criteria for the control group were as follows: aged 40 years and older, no clinical or laboratory symptoms of metabolic disorders, no family history of diabetes, and not related to other participants in the study.

Additionally, the study group included 279 people with metabolic syndrome (MetS) recruited between 2012 and 2017 at the Bashkir State Medical University Clinic. MetS was defined as fulfilling at least three of the following criteria: waist circumference greater than 102 cm (men) or 88 cm (women), blood pressure (BP) greater than 130/85 mmHg, fasting triglyceride (TG) level greater than 1.7 mmol/L, fasting high-density lipoprotein (HDL) cholesterol level less than 1.03 mmol/L (men) or 1.3 mmol/L (women), and fasting glucose (FG) greater than 5.6 mmol/L [32,33]. To reduce the probability of errors due to sample stratification, all study participants were selected from populations historically rooted in the Volga-Ural region of the Russian Federation. The ethnic origin (up to the third generation) and the presence or absence of a family history of diabetes for all participants were established by conducting direct interviews with the potential participants.

### 2.2. Ethics, Consent, and Permissions

The study was performed in accordance with the Helsinki Declaration. The study protocol was approved by the Local Ethical Committee of the Institute of Biochemistry and Genetics of Ufa Federal Research Centre of the Russian Academy of Sciences (IBG UFRC RAS), Ufa, Russia (Ufa, Protocol No 8, 14 March 2012). All participants provided written informed consent.

### 2.3. Anthropometric Measurements and Biochemical Assays

Anthropometric measurements were performed in accordance with the WHO guidelines [34]. Body weight was measured to the nearest 1 kg using a lever balance while the participants were wearing light indoor clothing. Height was measured to the nearest 1 cm using a stadiometer with the participants standing barefoot. Waist and hip circumferences were estimated to the nearest 1 cm using a tape measure. Waist circumference was assessed at the midpoint between the last rib and the iliac crest at the end of a normal expiration. Hip circumference was measured with the participants standing at the level of the largest lateral extension of the hips. Body mass index (BMI; kg/m^2^) was calculated as body weight (kg) divided by height squared (m^2^). The waist-to-hip ratio (WHR) was calculated by dividing waist circumference by hip circumference. BP was measured three times for each participant in both arms at 1 min intervals after 5 min of rest in the seated position with a standard sphygmomanometer, and the average of three consecutive measurements was taken as a reference. Phases I and V of Korotkoff sounds were identified as SBP and DBP, respectively [35]. Blood samples were collected after an overnight (12 h) fast and 2 h after the meal (for the 2 h postprandial test). Plasma glucose was measured by the glucose oxidase technique, and plasma insulin levels were measured by an electrochemiluminescence immunoassay (Cobas Integra, Roche, Basel, Switzerland). The homeostasis model assessment of insulin resistance (HOMA-IR) was calculated as (fasting insulin [μIU/mL] × fasting glucose [mmol/L])/22.5 [36]. HbA1c was measured by high-performance liquid chromatography (ADAMS A1c HA-8182, Arkray, Inc., Kyoto, Japan). Total cholesterol (TC), triglyceride (TG), high-density lipoprotein (HDL), and low-density lipoprotein (LDL) levels were measured via photometry (Olympus, Hamburg, Germany). C-reactive protein levels were measured via the chemiluminescent immunoassay IMMULITE 2000 (Siemens Medical Solutions Diagnostics, Deerfield, IL, USA). Tumour necrosis factor alpha was measured by ELISA (enzyme-linked immunosorbent assay) using the “Vector-Best” test system in Russia. Biochemical parameters (albumin, alanine aminotransferase, aspartate aminotransferase, gamma glutamyltransferase, creatinine, urea) in blood serum were determined using a Cobas Integra 400 plus biochemical analyser (Cobas Integra, Roche, Basel, Switzerland). The modified Ferriman–Gallwey score was used to assess hirsutism in female participants by summing hair growth in nine body areas (upper lip, chin, chest, arm, upper abdomen, lower abdomen, upper back, lower back, and thighs) scored from 1 (minimal terminal hairs present) to 4 (equivalent to a hairy man) [37]. The clinical characteristics of the study groups are shown in Table 1 and more detailed description is provided in Supplementary Tables S5 and S6.

### 2.4. Eating Behaviour

Eating behaviour was assessed in people with T2D and control individuals using the Dutch Eating Behaviour Questionnaire (DEBQ) [38]. The DEBQ categorizes individuals into three main patterns: emotional eating, external eating, and restraint eating [38]. Emotional eating pertains to the inclination to eat in response to emotions or stress rather than physical hunger. External eating involves responding to external food cues, such as the sight or aroma of food, which can lead to overindulgence in food-rich environments. Restraint eating encompasses deliberate dietary control and efforts to limit food intake, but this can sometimes backfire, resulting in episodes of overeating (known as binge eating) and weight fluctuations [39]. The questionnaire included 33 items to account for three eating styles: the Emotional Eating Scale (13 items), the External Eating Scale (10 items), and the Restraint Scale (10 items). The only reverse-keyed item is item 21 (“Do you find it hard to resist eating delicious foods?”). Responses are given on a 5-point Likert scale ranging from 1 “never” to 5 “very often”. The average score is calculated for each subscale by adding scores obtained from single items and dividing them by the number of items contained in one subscale. The DEBQ was translated into Russian by Yu.L. Savchikova [40].

### 2.5. Genotyping and Quality Control

Whole venous blood samples were obtained from each participant, stored at −4 °C, and used for total DNA extraction. DNA extraction and genotyping were performed using standard procedures as previously described [29,30,41,42,43,44,45,46,47]. Genetic variants were selected for the analysis based on the results of the Phenome-Wide Association Studies (PheWASs). The variants selected for the study included those associated with metabolic traits (cholesterol levels, fat mass, T2D) and related disorders, including inflammatory diseases and complications caused by T2D (Supplementary Table S7). Allelic discrimination was performed by real-time polymerase chain reaction (PCR) with a Bio-Rad CFX96 (Bio-Rad Laboratories, Inc., Hercules, CA, USA) using TaqMan SNP genotyping assays (Thermo Fisher Scientific, Waltham, MA, USA). For quality control, 5% of the genotyped samples were randomly selected for regenotyping, and all the newly obtained results were identical to the previously determined genotyping data.

### 2.6. Association Analysis

Associations between the studied loci and eating behaviour, clinical parameters, T2D status, and MetS status were explored by linear or logistic regression analysis under the additive genetic model adjusted for age and sex with PLINK 1.9 [48]. The additive genetic model assumes that having two risk alleles has twice the impact on the outcome compared to carrying one risk allele. Given that many of the examined biomarkers (glucose, HbA1c, lipids, blood pressure, etc.) can be influenced by specific treatments, we performed the adjustment for medication status accordingly. Specifically, individuals undergoing glucose-lowering therapy were excluded from the analysis, those undergoing lipid-lowering therapy had their LDL values adjusted by dividing by 0.7, TC adjusted by dividing by 0.8, and HDL adjusted by dividing by 1.05 [49]. Individuals taking antihypertensive medications had their blood pressure levels adjusted by adding 10 mmHg and 15 mmHg to their diastolic blood pressure (DBP) and systolic blood pressure (SBP), respectively [50]. Sex-specific biomarkers (such as testosterone and Ferriman–Gallwey score) were analysed separately in the relevant sex groups. All traits exhibiting non-normal distribution underwent log-transformation. We applied the Benjamini–Hochberg procedure to control for the expected ratio of false-positive classifications (false discovery rate—FDR) [51]. P_FDR_ values less than 0.05 were considered significant.

### 2.7. Mendelian Randomization

**Figure 1:**
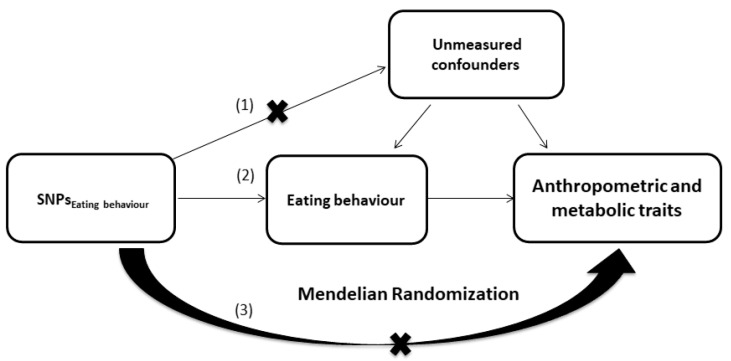
Two-sample Mendelian randomization. MR relies on the following three core assumptions: (1) the instrument is independent of measured and unmeasured confounders of the association between the exposure (eating behaviour measured with DEBQ) and outcome (metabolic syndrome and 17 anthropometric and metabolic parameters in people with metabolic syndrome); (2) the genetic variant(s) being used as an instrument is associated with the exposure; and (3) there is no independent pathway between the instrument (SNPs for eating behaviour) and outcome (metabolic syndrome and 17 anthropometric and metabolic parameters in people with metabolic syndrome) other than through the exposure (eating behaviour)—otherwise known as horizontal pleiotropy or the exclusion restriction assumption [52].

To assess causality between eating behaviour and metabolic traits, we performed two-sample MR with eating behaviour patterns as the exposure and MetS and clinical parameters of MetS patients as an outcome using two independent datasets (Figure 1). Subsequently, we performed MR analysis to evaluate the causal relationship between eating behaviour and MetS and other cardiometabolic traits using openly available summary statistics data from published genome-wide association studies (Supplementary Table S6).

The single-nucleotide polymorphisms (SNPs) significantly associated with emotional eating (rs6313 at the *HTR2A* gene locus) and external eating (rs623988 at *HTR1D* and rs9295474 at CDKAL1) or their proxies (rs604030 for rs623988, r^2^ = 1.0; rs2206739 for rs9295474, r^2^ = 0.986; Supplementary Table S6) were used as genetic instruments for the MR analyses. Causal effects estimated via MR are valid only if the following core assumptions hold true: (1) the genetic instrument has a true effect on the exposure, (2) it affects the outcome through its effect on the exposure, and (3) it is independent of any measured and unmeasured confounding factors of the exposure–outcome relationship. Summary statistics used for the MR analyses included UK Biobank data (height, weight, waist circumference, hip circumference, BMI, total cholesterol (TC), triglyceride (TG), systolic blood pressure (SBP), diastolic blood pressure (DBP), and HbA1c), obtained from http://www.nealelab.is/uk-biobank/, accessed on 1 February 2024). The GWAS summary statistics for MetS in the UK Biobank participants were assessed via [53]. The data on glycaemic traits (FG, FI, 2 h glucose, and HOMA-IR) were obtained from MAGIC investigators and were downloaded from www.magicinvestigators.org [54]. The HDL and LDL data were obtained from the Global Lipids Genetics Consortium [55]. For the WHR, we used summary statistics data from the GIANT Consortium [56]. The data on the SNPs included for each trait are provided in Supplementary Table S6. All MR analyses were performed using the MRCIEU/TwoSampleMR R software package version 0.5.7 [57]. We utilized the Strengthening the Reporting of Observational Studies in Epidemiology Using MR (STROBE-MR) approach to ensure the clarity and transparency of the reporting of our results.

## 3. Results

### 3.1. Association Analysis

We examined the associations between genetic variants in 26 genes involved in neurotransmitter signalling and oxidative stress/chronic inflammation pathways and eating behaviour patterns in people with T2D (*n* = 295) from the Volga-Ural region of the Eurasian continent using linear regression analysis with the additive genetic model adjusted for age and sex. We found that *HTR1D* rs623988 and *CDKAL1* rs9295474 polymorphisms were associated with external eating, while *HTR2A* rs6313 was associated with emotional eating (Table 2, Supplementary Table S1).

Logistic regression analysis adjusted for age and sex was conducted for the people with T2D and healthy controls (*n* = 597), and the results revealed that *HTR2A* rs6313, *NPY2R* rs1047214, *HTR1F* rs56398417, *HTR3A* rs1062613, *HTR2C* rs6318, and CXCR2 rs2230054 were associated with T2D. Restrained eating was suggested to be (at *p* < 0.05) associated with the *HTR1D* rs623988, *BDNF* rs11030107, *MC4R* rs17782313, and *CXCL8* rs4073 genetic variants, but these associations did not reach significance after Benjamini–Hochberg adjustment for multiple testing (Supplementary Table S1).

Further analyses of a second sample group of people with MetS (*n* = 279) and controls (*n* = 397) revealed significant associations between MetS and polymorphic loci in the *CRP* (rs2794521), *ADCY3* (rs17799872), *GHRL* (rs696217), *CDKAL1* (rs9295474), *BDNF* (rs11030107), *CHRM4* (rs2067482), *CHRM1* (rs2067477), *HTR3A* (rs1062613), and *AKT1* (rs3803300) genes (Table 2). Moreover, the genetic variant in the *HTR2C* gene (rs6318) was associated with height, BMI, and waist circumference; *ADCY3* (rs17799872) with BMI; and *SIRT1* rs3758391 with the WHR, in people with MetS (Table 2). The *CDKAL1* variant (rs9295474) was significantly associated with the serum ALB concentration (P_FDR_ = 0.02) (Table 2); suggestive associations (at *p* < 0.05) detected between this polymorphism and other metabolic parameters (CRP, fibrinogen, K^+^, uric acid), including glycaemic traits (such as FG, HbA1c, and HOMA-IR), did not survive adjustment for multiple testing (Supplementary Table S2).

### 3.2. Mendelian Randomization

**Figure 2:**
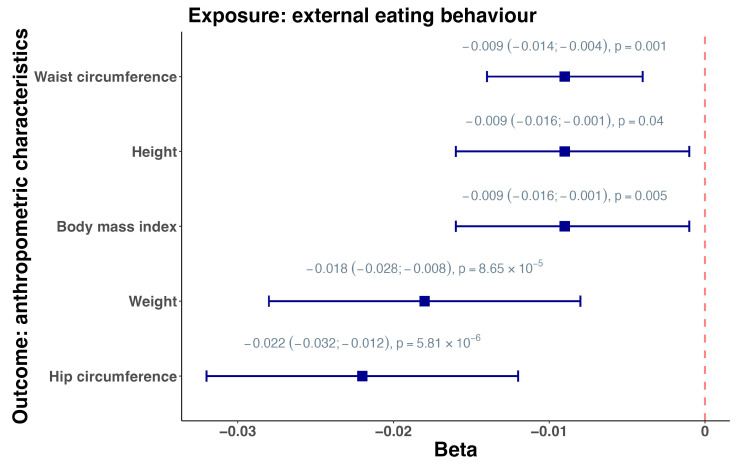
Forest plot visualizing the significant results of Mendelian randomization analysis between external eating (exposure) and anthropometric characteristics using genome-wide data. Beta coefficients with 95% confidence intervals and significance values are provided.

We conducted two-sample MR analysis using the group of people with T2D for exposure evaluation and the group of people with MetS and the control group for the outcome assessment. The results showed that external eating behaviour was inversely associated with glycated haemoglobin (HbA1c) in people with MetS (beta = −0.347, SE = 0.158, *p* = 0.016) (Supplementary Table S3). Next, we performed two-sample MR using genome-wide summary statistics from the UK Biobank (BMI, cholesterol, SBP, DBP, HbA1c, height, hip circumference, waist circumference, weight, triglycerides), the meta-analyses of glucose and insulin-related traits consortium or MaGIC (2 h glucose, fasting glucose, fasting insulin, HOMA-IR), the Global Lipids Genetics Consortium or GLGC (LDL), and the Genetic Investigation of Anthropometric Traits or GIANT Consortium (waist–hip ratio). The results demonstrated that external eating was also associated with BMI (beta = −0.009, SE = 0.008, *p* = 0.005), height (beta = −0.009, SE = 0.008, *p* = 0.040), hip circumference (beta = −0.022, SE = 0.010, *p* = 5.81 × 10^−6^), waist circumference (beta = −0.009, SE = 0.005, *p* = 0.001), and weight (beta = −0.018, SE = 0.010, *p* = 8.54 × 10^−6^) (Figure 2, Supplementary Table S4).

## 4. Discussion

We investigated the impact of distinctive eating behaviour patterns on metabolic traits. Our primary focus was on 26 genes that fall within the domains of neurotransmitter signalling and oxidative stress/chronic inflammation pathways. We found associations between *HTR2A* rs6313 and emotional eating, as well as *HTR1D* rs623988 and *CDKAL1* rs9295474 with external eating (Table 1). *CDKAL1* rs9295474 exhibited strong association with T2D in multi-ethnic cohorts from Southeast Asia [58], with its polymorphisms potentially affecting insulin resistance in response to varying levels of dietary fat and protein intake [59]. Moreover, *CDKAL1* rs9295474 was notably associated with hypertension SBP and DBP in individuals of European ancestry [60]. Intriguingly, according to our data, *CDKAL1* rs9295474 was associated with albumin (beta = −1.48, P_FDR_ = 0.02), which may have implications for future treatments as albumin is a proposed drug carrier for neuromedin U, a neuropeptide involved in the regulation of food intake, with a powerful anorexigenic ability [61]. Decreased levels of albumin were linked to increased food intake, inflammation, and obesity, potentially due to its ability to bind ghrelin, thus implicating it in the appetite regulation [62,63,64]. *HTR2A* rs6311 was associated with anorexia nervosa and binge eating disorder [65,66], while *HTR1D* SNPs were linked with anorexia nervosa [67].

We subsequently explored the causal relationship between eating behaviour patterns and metabolic traits utilizing the robust framework of MR. This analysis was initially conducted on two distinct cohorts of individuals from the Volga-Ural region of Eurasia, as delineated in Supplementary Table S4, and further extended to include a broader perspective through the utilization of summary statistics gleaned from GWASs, as also detailed in Supplementary Table S4.

Previous research links external eating to insulin resistance, while restraint and emotional eating predict obesity and overweight in adolescents [68]. Our findings showed a compelling causal relationship between external eating and glycaemic traits, namely, HbA1c, which was particularly pronounced within the Volga-Ural sample. Furthermore, we observed a noteworthy influence of external eating on various anthropometric phenotypes within the GWAS cohorts. These results were consistent with the prior literature documenting associations between the three DEBQ subscales and weight-related characteristics, including weight at 20 years, weight in adulthood, and BMI [69,70,71,72]. A counterintuitive inverse relationship between external eating and weight and BMI has also been found in population-based studies of adolescents [73] and in adults with long-standing type 2 diabetes [74]. However, the link between external eating and BMI is less clear in obese individuals and healthy adults. A recent meta-analysis failed to ascertain significant correlations between external eating and BMI [75]. The BMI polygenic score was previously shown to be negatively associated with external eating [76]. A possible explanation for these findings is that either parents limit the amount and type of food available to their teenaged children or that they control their weight with compensatory behaviours such as physical activities [77]. Regarding people with diabetes, functional magnetic resonance imaging (fMRI) studies have shown increased responses to depicted foods in the frontal cortex and insula compared to participants without diabetes. This frontal brain activity was associated with external eating, as well as dietary self-efficacy and self-care, suggesting that as a result of the need to follow a life-long restrictive diet, people with diabetes have developed greater cognitive control over their food intake [74]. It has also been suggested that uncontrolled eating acts as an intermediate phenotype explaining the link between broad psychological constructs and food intake/BMI [78].

Interestingly, in our study, we could not detect any causal relationship between emotional eating and the studied metabolic parameters. This observation held true both within the Volga-Ural sample and when extrapolating our findings using GWAS summary statistics. This is intriguing, given prior hypotheses suggesting that emotional eating precedes external eating, which, in turn, contributes to weight gain [71].

In the context of our research, we analysed the causal relationships between quantitative eating traits (emotional eating, external eating, and dietary restraint) measured in people with T2D and unaffected individuals and between metabolic and anthropometric characteristics in people with MetS. Study limitations include the particular composition of our study dataset that may have introduced certain confounding variables into our analyses. Moreover, we recognize that the causal relationships elucidated in our study may manifest differently in a broader-population-based cohort. In particular, the causal relationship between external eating and Hba1C detected in the Volga-Ural study sample might reflect the effect of glucose-lowering drugs. The somewhat limited sample size, especially in the T2D groups, may have constrained our ability to detect associations between eating behaviour and variants with smaller effect sizes. As such, we emphasize that the generalization of our study results to other populations necessitates rigorous validation through independent replication efforts.

Study strengths include the novelty of genetic loci associated with eating behaviour patterns, assessed using the DEBQ, in people with T2D and healthy individuals, as well as the application of a robust the two-sample MR approach to explore the potential causal links between eating behaviour patterns and a wide range of metabolic traits in an independent sample of individuals with MetS. Our study benefits from utilizing our own data from the Volga-Ural region of Eurasia, as well as GWAS results from large consortia such as the MaGIC, GLGC, GIANT Consortium, and the UK Biobank.

Our study’s findings offer insight into the biological mechanisms connecting eating behaviour with metabolic disorders like diabetes, potentially serving as a basis for developing strategies to enhance metabolic health. A notable advantage of this approach is the stability of molecular genetic markers throughout an individual’s life, enabling their use from birth onward.

## 5. Conclusions

Using a Mendelian randomization approach, we demonstrated a strong causal relationship between external eating and glycaemic traits (HbA1c) in individuals from the Volga-Ural region of Eurasia. Using genome-wide data, we established that external eating influenced various anthropometric traits, including height, weight, body mass index, and waist and hip circumference. Subsequent investigations are warranted to unveil the molecular mechanisms underpinning the observed relationships.

## Figures and Tables

**Table 1:** Clinical characteristics of the study group.

Parameter	All			Men			Women		
	Control (N = 397)	T2D (N = 200)	MetS (N = 279)	Control (N = 201)	T2D (N = 57)	MetS (N = 80)	Control (N = 196)	T2D (N = 143)	MetS (N = 199)
Age (years)	49.65 ± 10.88	61.49 ± 9.55	57.01 ± 6.97	47.24 ± 11.06	60.44 ± 9.78	56.19 ± 7.50	52.12 ± 10.13	61.92 ± 9.46	57.35 ± 6.73
External Eating	3.33 ± 1.02	3.54 ± 0.74	NA	3.09 ± 1.07	3.35 ± 0.55	NA	3.48 ± 0.96	3.62 ± 0.79	NA
Emotional Eating	2.64 ± 1.27	4.46 ± 1.1	NA	2.18 ± 1.3	4.24 ± 1.07	NA	2.92 ± 1.16	4.54 ± 1.1	NA
Restraint	2.86 ± 1.13	2.89 ± 0.75	NA	2.56 ± 1.28	2.83 ± 0.86	NA	3.05 ± 0.98	2.91 ± 0.71	NA
Height (cm)	173.26 ± 8.21	161.7 ± 7.96	169.43 ± 7.23	176.32 ± 6.25	168.79 ± 6.76	175.72 ± 4.57	163.24 ± 5.41	158.87 ± 6.53	166.90 ± 6.53
Weight (kg)	79.38 ± 13.09	80.97 ± 15.46	89.29 ± 5.46	82.79 ± 12.12	86.82 ± 16.19	93.06 ± 5.95	68.20 ± 9.46	78.64 ± 14.57	87.77 ± 4.42
BMI (kg/m^2^)	27.68 ± 4.5	30.92 ± 5.25	31.17 ± 2.45	27.39 ± 4.26	30.41 ± 5.07	30.11 ± 2.00	27.98 ± 4.73	31.12 ± 5.32	31.60 ± 2.49
Cholesterol (mmol/L)	5.09 ± 0.64	5.43 ± 1.14	5.88 ± 0.71	5.17 ± 0.55	5.57 ± 0.95	5.91 ± 0.72	5.06 ± 0.67	5.38 ± 1.21	5.86 ± 0.71
Triglycerides (mmol/L)	1.48 ± 0.6	1.68 ± 1.33	1.72 ± 0.51	1.48 ± 0.61	1.93 ± 1.45	1.74 ± 0.47	1.48 ± 0.60	1.58 ± 1.28	1.71 ± 0.53
HDL (mmol/L)	1.09 ± 0.37	1.2 ± 0.51	1.01 ± 0.13	1.07 ± 0.34	1.27 ± 0.58	0.96 ± 0.10	1.09 ± 0.38	1.17 ± 0.48	1.02 ± 0.13
LDL (mmol/L)	2.96 ± 1.08	3.05 ± 1.43	3.17 ± 0.19	3.03 ± 0.98	3.18 ± 1.57	3.23 ± 0.16	2.93 ± 1.11	3 ± 1.37	3.15 ± 0.20
HbA1c (%)	4.89 ± 0.6	7.48 ± 0.99	5.28 ± 1.00	4.87 ± 0.65	7.47 ± 0.94	5.12 ± 0.91	4.89 ± 0.58	7.48 ± 1.01	5.34 ± 1.03
Fasting Glucose (mmol/L)	4.88 ± 0.71	7.22 ± 1.95	5.33 ± 1.38	4.79 ± 0.66	7.2 ± 2	5.19 ± 1.31	4.90 ± 0.73	7.22 ± 1.93	5.38 ± 1.41
2 h glucose (mmol/L)	NA	9.93 ± 2.2	6.58 ± 2.42	NA	10.17 ± 2.36	6.35 ± 2.48	NA	9.83 ± 2.13	6.67 ± 2.40
C-peptide (ng/mL)	2.31 ± 0.94	2.65 ± 5.39	NA	2.39 ± 0.87	2.18 ± 0.94	NA	2.28	2.83 ± 6.34	NA

T2D—type 2 diabetes, MetS—metabolic syndrome, N—sample size, BMI—body mass index, LDL—low-density lipoproteins, HDL—high-density lipoproteins, HbA1c—glycated haemoglobin. Data are presented as mean values ± standard deviation.

**Table 2:** Significant associations between the studied loci and eating behaviour patterns and metabolic traits.

Gene	SNP	EA	NEA	EAF	N	Beta/OR	SE	P	P_FDR_
Emotional eating									
*HTR2A*	rs6313	A	G	0.47	286	0.36	0.11	0.001	0.041
External eating									
*HTR1D*	rs623988	A	G	0.29	295	0.32	0.08	1.20 × 10^−4^	3.60 × 10^−3^
*CDKAL1*	rs9295474	C	G	0.64	294	0.22	0.08	0.003	0.047
Type 2 diabetes									
*CXCR2*	rs2230054	T	C	0.44	595	1.8	0.15	8.87 × 10^−5^	8.87 × 10^−4^
*HTR1F*	rs56398417	C	T	0.84	597	2.61	0.24	5.01 × 10^−5^	7.52 × 10^−4^
*NPY2R*	rs1047214	T	C	0.62	400	1.82	0.17	4.56 × 10^−4^	3.42 × 10^−3^
*HTR3A*	rs1062613	T	C	0.19	596	2.13	0.18	4.03 × 10^−5^	7.52 × 10^−4^
*HTR2A*	rs6313	A	G	0.47	572	1.58	0.15	0.002	0.012
*HTR2C*	rs6318	C	G	0.09	593	2.07	0.24	0.002	0.012
Metabolic syndrome									
*CRP*	rs2794521	C	T	0.21	621	6.64	0.17	4.83 × 10^−28^	1.45 × 10^−26^
*ADCY3*	rs17799872	A	G	0.09	641	1.90	0.19	0.001	0.004
*GHRL*	rs696217	T	G	0.08	639	2.20	0.18	2.02 × 10^−5^	1.51 × 10^−4^
*CDKAL1*	rs9295474	G	C	0.36	634	1.70	0.13	4.26 × 10^−5^	2.56 × 10^−4^
*BDNF*	rs11030107	G	A	0.13	627	1.91	0.17	1.23 × 10^−4^	0.001
*CHRM4*	rs2067482	T	C	0.08	641	0.45	0.28	0.005	0.017
*CHRM1*	rs2067477	A	C	0.04	637	3.33	0.26	3.80 × 10^−6^	3.80 × 10^−5^
*HTR3A*	rs1062613	T	C	0.19	636	2.10	0.16	2.53 × 10^−6^	3.79 × 10^−5^
*AKT1*	rs3803300	A	G	0.03	637	2.48	0.31	0.003	0.012
Height									
*HTR2C*	rs6318	C	G	0.09	242	−3.60	0.89	7.32 × 10^−5^	0.002
Body mass index									
*ADCY3*	rs17799872	A	G	0.09	245	1.13	0.30	1.90 × 10^−4^	0.003
*HTR2C*	rs6318	C	G	0.09	242	1.53	0.34	1.28 × 10^−5^	3.83 × 10^−4^
Waist circumference									
*HTR2C*	rs6318	C	G	0.09	242	4.88	1.36	3.90 × 10^−4^	0.012
Waist–hip ratio									
*SIRT1*	rs3758391	C	T	0.52	234	0.01	0.00	0.001	0.040
Albumin									
*CDKAL1*	rs9295474	G	C	0.36	237	−1.48	0.43	0.001	0.020

SNP—single-nucleotide polymorphism; EA—effect allele; NEA—non-effect allele; EAF—effect allele frequency; N—sample size; Beta—effect size (for emotional and external eating, height, BMI, waist circumference, WHR, and albumin); OR—odds ratio (for T2D and MetS); SE—standard error; P—level of significance; P_FDR_—level of significance with the Benjamini–Hochberg adjustment.

## Data Availability

The original contributions presented in the study are included in the article/Supplementary Material, further inquiries can be directed to the corresponding author.

## Supplementary Materials

The following supporting information can be downloaded at https://www.mdpi.com/article/10.3390/nu16081166/s1, Supplementary Table S1: Associations of the studied loci with the patterns of eating behaviour and type 2 diabetes, Supplementary Table S2: Associations of the studied loci with metabolic syndrome and related traits, Supplementary Table S3: Causal relationship between eating behaviour patterns and anthropometric and cardiometabolic phenotypes identified by Mendelian Randomization, Supplementary Table S4: Causal relationship between eating behaviour pat-terns and anthropometric and cardiometabolic phenotypes identified by Mendelian Randomization using genome-wide data, Supplementary Table S5: Clinical characteristics of people with metabolic syndrome and the control group, Supplementary Table S6: Clinical characteristics of people with type 2 diabetes and the control group, Supplementary Table S7: The list of studied loci, Supplementary Table S8: Summary statistics for the loci significantly associated with eating behaviour in genome-wide associated studies for metabolic disorders.
